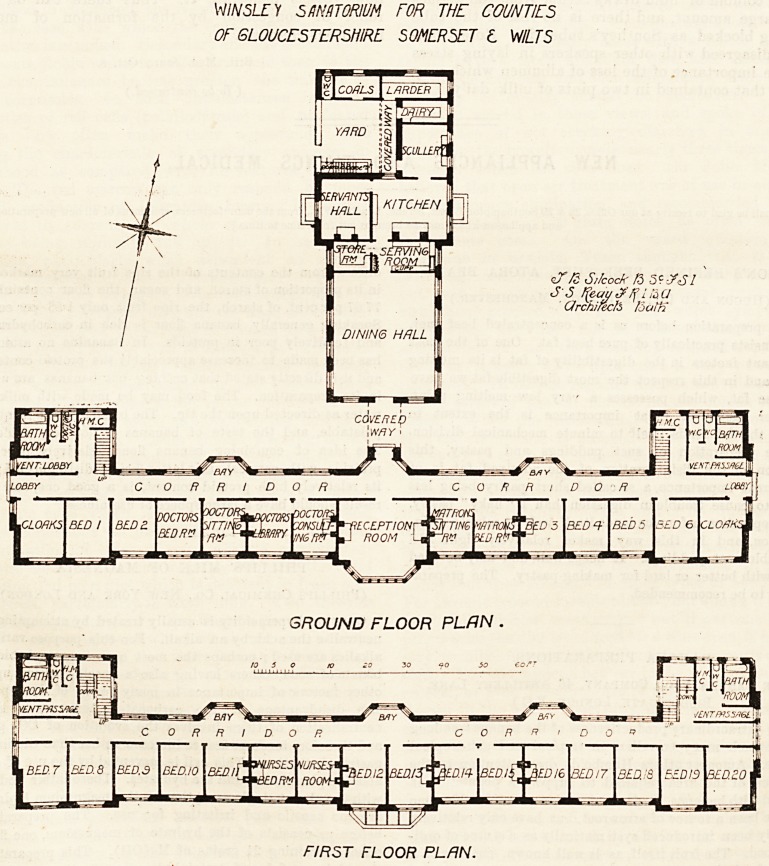# The Winsley Sanatorium for Consumptives at Limpley Stoke, near Bath

**Published:** 1905-01-28

**Authors:** 


### HOSPITAL ADMINISTRATION.

### CONSTRUCTION AND ECONOMICS.

### THE WINSLEY SANATORIUM FOR CONSUMPTIVES AT LIMPLEY STOKE, NEAR BATH.

THE memorial stone of this building was laid early in 1903 by Lady Dickson Poynder, and it was formally opened in January 1905. About the time of the laying of the foundation-stone the financial position of the sanatorium was stated by Dr. Watson Williams to be as follows:— Subscriptions and donations amounted to £5,571, and promises of further help to the amount of £4,191 had been received. The purchase of the site, the levelling thereof, and some other expenses amounted to fully £3,000. The tender for the erection of the building was £6,877.

The plan of the sanatorium proper is linear in shape; and it is only two stories in height except at the ends, which rise to a height of three stories, by which the façade of the building is greatly improved, and accommodation is provided for the domestic staff.

The sanatorium faces almost due south. On the ground floor the centre is taken up by a fine reception-room having a large bay window. On the east side of the reception-room are the matron's sitting-room and bedroom; and on the west side are the medical officer's consulting-room, library, sitting-room, and bedroom. Further east than the matron's rooms are bedrooms for four patients, and further west than the medical officer's rooms are bedrooms for two patients and a cloak-room. Running behind all these rooms is a handsome corridor, with abundance of light and air. This corridor has three bay windows and eight other widows. It is about 195 feet long, 6 feet wide, and has an entrance at each end, adjoining which are the bathrooms and closets. The latter are separated from the main corridor by a ventilating passage, and a staircase is incorporated with each of these sanitary blocks. At the centre of the main corridor is a short covered way, which gives access to the dining-hall and kitchen, the latter being surrounded by its offices. All this part is very well designed.

The first-floor plan is similar to that of the ground floor save that the space above the reception-room is used to provide accommodation for two nurses, and as none of the superior officers sleep on this floor there are rooms for 14 patients, making a total of 20 patients' bedrooms in the sanatorium. Each of these rooms is 13 feet 6 inches long, 11 feet 6 inches wide, 10 feet high. The windows take up nearly the whole of the south end, and are of the casement pattern; each section, both above and below the transom, is made to open outwards. The opposite or corridor end of the room is provided with a long casement above the door-head, opening on pivots, thus ensuring thorough cross-ventilation. Some of the rooms are warmed by open fireplaces, and the whole building is supplied with hot-water radiators acting on the low-pressure system. The dining-hall has very properly been made of large size, and it will accommodate 60 patients. It is also well lighted and ventilated, and the serving-rooms, store-rooms, etc., are admirably arranged. Inside, the walls of all the rooms are covered with one of the hard-setting cements, and the angles and corners have been rounded off. Externally the building is faced with a white cement, and the roofs are covered with red tiles.

Speaking of the plan from a general point of view it must be pronounced good, but we are not quite pleased to note that two-thirds of the ground floor to the south are given up to officers' rooms and cloak-rooms. It would certainly have been better to provide accommodation for these officers elsewhere, and reserve the whole of the south front for patients, more especially as there would seem to be no provision made for the treatment of any of the patients in huts entirely detached from the main building. We regret this omission, and as time goes on it will certainly be demonstrated by experience that a sanatorium for consumptives should be chiefly composed of these huts, with little more than an administrative centre in the background, If sufficient land be possessed by the managers of the Winsley Institution the building will be admirably adapted for this development.

Other things we miss are verandahs. Some patients must be confined to bed for many days at a time, and it would relieve some of the dulness inseparable from this if they could have their beds wheeled on to a verandah for a few hours every day.

The architects were Messrs. Silcock and Reay, of Bath, and the contractors were Messrs. Jacob Long and Sons, also of Bath.

## Figures and Tables

**Figure f1:**